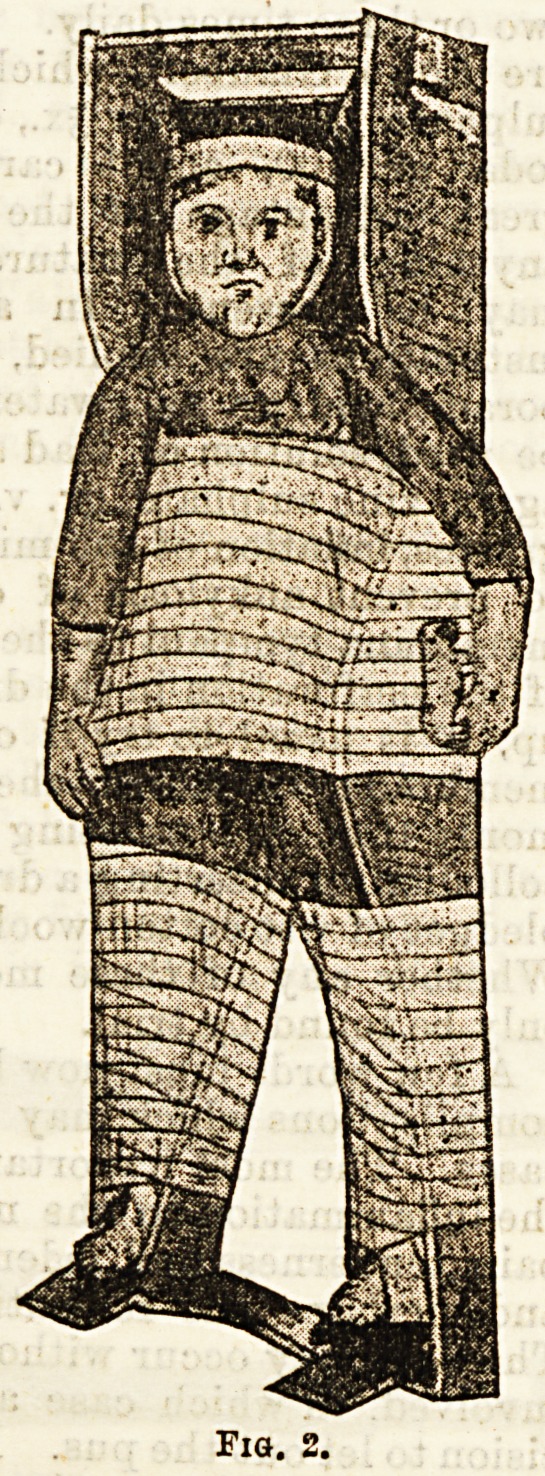# The Treatment of Spinal Disease

**Published:** 1892-12-03

**Authors:** 


### The Hospital Clinic.

[*The Editor will be glad to receive offers of co-operation and contributions from members of the profession. All letters should be addressed to* The Editor, The Lodge, Porchester Square, London, W.]

### PADDINGTON GREEN CHILDREN'S HOSPITAL.

### The Treatment of Spinal Disease.

The difficulties in carrying out the prolonged treatment necessary in cases of spinal disease are well known, and a short account of the methods in use at Paddington Green Children's Hospital may be of value to our readers. The treatment has not been devised for use in the wards, but for those out-patients who require absolute rest for a prolonged period, and who have only their home nursing to depend on. The method to be described is one which secures complete fixation of the whole body, and which possesses the additional advantages of being comfortable and economical.

The splint, which is known as Phelps' box (Fig. 1), is a trough of wood in which the patient lies, with a broad floor for the body and two diverging parts for the lower limbs. Opposite the buttocks is a hollowed out part, so that defaecation and micturition can be carried out without moving the patient out of the box. In the construction of the box, for the length, the height of the child is measured, and twelve inches are allowed

Fig. 1.

Fig. 2.

at the head and eight inches at the foot for the growth of the patient, and the application of extension; for the width, the breadth at the hips and shoulders is taken, and two or three inches are then allowed at each side for comfortable padding. The sides of the box are about six inches high at the upper part, and decrease as they pass down to the feet. They are hollowed out opposite the shoulders, so as to allow of the free movement of the arms. A small ridge of wood runs along the inside of each leg piece. The head is formed of two strips of wood, with a space between for the passage of the extension apparatus. At the feet there are two perforated vertical pieces of wood, to which the feet may be bandaged, with an intervening pad, or through which an extension apparatus may pass. Opposite the heels the wood is hollowed out so as to prevent any undue pressure at that part. The floor and sides of the box are padded with cushions of horse-hair, covered with American cloth, and for which the child's mother is instructed to make covers of white cloth (pillow slips). There are three of those pads for the trunk and head, one for each leg, and one for each side, and all are shaped to fit exactly the part they cover. These splints, with the padding, are made for the hospital by an ordinary carpenter, and the price is from eighteen to twenty-one shillings. If there is a marked spinal curvature, the supporting pad must be made so as to avoid all pressure at that part, and this is done by the nurse. The patient, with a nightdress as the sole clothing, is then placed in the box and securely bandaged in. (Fig. 2.) The most suitable nightdress is of flannel, which encircles the trunk and arms in the usual way, and is continued to the ankles in the form of two broad strips, which are simply tucked in around the limbs when the child is in the box. Extension strapping, with tapes attached, is then applied to the legs in the ordinary manner, and the tapes are passed through the holes in the footpiece, and tied there. The head is then fixed by webbing passing round the forehead, and under the occiput, with a connecting band under the chin. To this is attached on each side, and well back so as to make traction in the line of the spine, and not merely tilt up the face, a piece of webbing which passes through the head of the box, and is fixed to a buckle on the outside. Each lower limb is then bandaged from the ankle to the upper part of the thigh, and the trunk is bandaged from the level of the iliac crests to the armpits, the corresponding part of the box being included. The patient is now firmly fixed in position, his powers of movement being limited to the arms. His face, arms, and legs can be washed without moving him out of the box by undoing the bandages, but his mother is instructed to take him out once a week for a thorough ablution. For this purpose the apparatus may be turned upside down on a bed, and then lifted off the child, but practically it is better to teach the patient to hold himself rigid, which is easily done, and then the nurse, placing one hand under the buttock, and the other under the shoulders, lifts him out on to the bed. It is found by experience that the patients soon get accustomed to their narrow sphere, and there are some now attending the hospital who have been in their boxes for over two years. For children under two years of age the treatment is not so suitable, and a double Thomas' splint is preferred.

There are certain cases in which the above treatment is not applicable, notably those in which paralysis, or abscess formation is present. If there is paralysis associated with spinal disease, the patient is placed in the horizontal position in bed, sand bags are used to fix the trunk and lower limbs, and extension is applied to the head and legs. The extension is by means of pulleys and weights, the latter varying from three to five pounds. The horizontal position takes off the weight of the upper part of the body, and the extension overcomes the muscular contraction, which is the second factor in the production of spinal deformity. By these means many cases of spinal paralysis, for which laminectomy appeared to be the only hope, have been completely cured. When the paralytic symptoms have passed off the patient must be treated by prolonged rest, and for this Phelps' box is employed.

Spinal abscesses are opened, irrigated, injected with iodoglycerine, and then stitched up. Antiseptic precautions are used throughout the operation. The skin is first washed with soap and water, and then scrubbed with "strong solution," of which the formula is as follows: Corrosive sublimate, one part; carbolic acid lotion (1-20), 500 parts.

The opening varies in position with the site of the abscess, and is not made with a view to drainage, which is not employed. It must be sufficiently large to allow of the entrance of the nozzle of the irrigator and the free exit of the lotion at the same time. Irrigation is carried out with warm sublimate solution (one part in ten thousand), the lotion being contained in a glass jar fixed to the wall, with rubber tubing attached. The wall of the abscess cavity is scraped with a piece of rough sponge, the finger, or a sharp spoon, and all the *débris* is swept out by irrigation. The abscess cavity is then emptied and dried, and from one to two ounces of a mixture containing iodoform (ten parts) and glycerine (ninety parts) are injected by means of a glass syringe. The wound is carefully stitched up with catgut sutures, and a dressing of double cyanide gauze and salicylic wool is applied. These cases frequently heal by first intention, and give no further trouble, but should the wound break down, the process is repeated, and in addition the superficial parts of the sinus are carefully dissected away. When a large superficial abscess is present, it is laid freely open and cleansed. The sinus leading to this through the deep muscles is found, and the deeper abscess is treated by scraping and irrigation. The skin wound is then stitched up in its whole extent. In all cases of operation for spinal abscess the after treatment is considered most important, and consists in absolute fixation in bed with double extension, as described above in connection with spinal paralysis.

The local treatment of spinal disease is, of course, always accompanied by constitutional treatment, comprising nourishing food, tonics, and, if possible, a prolonged stay in the country, or at the seaside.

## Figures and Tables

**Fig. 1. f1:**
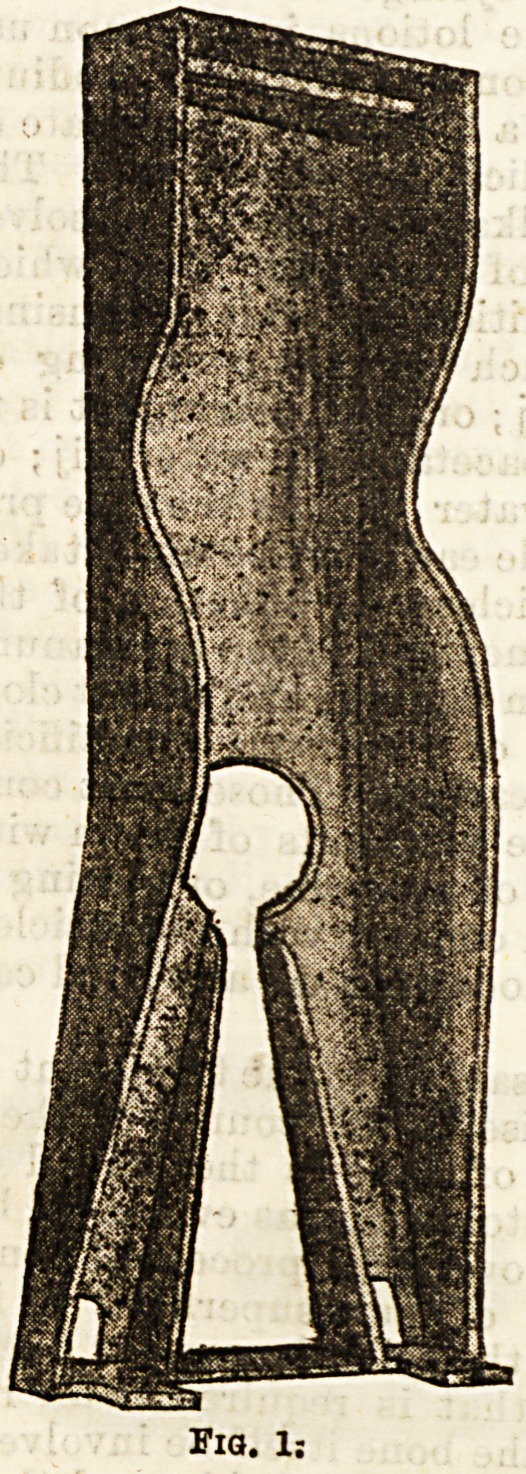


**Fig. 2. f2:**